# Dual anticoagulant and antiplatelet effects of fructose-1,6-diphosphate in vitro

**DOI:** 10.1038/s41598-025-30815-7

**Published:** 2026-01-05

**Authors:** Yalong Zhang, Xingguo Zhong, Lin Zhou, Yuan Fang, Tongqing Chen

**Affiliations:** 1 Pharmacy Department, Guoyang Hospital of Traditional Chinese Medicine, No. 500 Zhabei Road, Guobei Street, Guoyang, Bozhou, 233600 Anhui China; 2 General Surgery Department, Anhui No. 2 Provincial People’s Hospital, No. 1868 Dangshan Road, Hefei, 230041 Anhui China; 3 Medical Record and Statistics Department, Anhui No. 2 Provincial People’s Hospital, No. 1868 Dangshan Road, Hefei, 230041 Anhui China; 4 Blood Transfusion Department, Anhui No. 2 Provincial People’s Hospital, No. 1868 Dangshan Road, Hefei, 230041 Anhui China; 5 Anhui No.2 Provincial People’s Hospital Clinical College, Anhui Medical University, No. 1868 Dangshan Road, Hefei, 230041 Anhui China

**Keywords:** Fructose-1,6-diphosphate, Thromboelastography, Coagulation factors, Platelet aggregation, Anticoagulation, R-time, Cardiology, Diseases, Drug discovery, Medical research

## Abstract

While fructose-1,6-diphosphate (FDP) has been clinically applied in ischemic conditions, its comprehensive effects on hemostasis remain to be fully elucidated. This in vitro study examined the influence of FDP on coagulation and platelet function using thromboelastography (TEG) across three platforms, coagulation factor assays, and platelet aggregation tests over a concentration range of 0–6 mg/mL. The results revealed that FDP concentration-dependently prolonged TEG R-time (*P* < 0.01), with an increase of 21.8–48.3% at the clinically relevant concentration of 3.71 mg/mL. Strong inverse correlations were observed between FDP concentration and the activities of factors V, VII, IX, XI, and XII (*r *= − 0.989 to − 0.997, *P* < 0.001), whereas factors II, VIII, and X remained unaltered. Furthermore, FDP significantly inhibited platelet aggregation (*P* < 0.001), nearly abolishing epinephrine- and ADP-induced aggregation at 6 mg/mL under unbuffered conditions. pH-control experiments confirmed that the anticoagulant effects were FDP-specific, while the antiplatelet effects were primarily mediated by FDP with a partial pH-dependent component. These findings demonstrate that FDP possesses dual anticoagulant and antiplatelet properties through selective inhibition of coagulation initiation factors and broad suppression of platelet responsiveness, suggesting potential implications for clinical use in high-risk populations and warranting further investigation.

## Introduction

Fructose-1,6-diphosphate (FDP), a key intermediate in the glycolytic pathway, has been clinically applied for several decades in the treatment of ischemic disorders, including myocardial infarction and hemorrhagic shock. Its therapeutic efficacy in these contexts is primarily attributed to its cytoprotective effects and ability to enhance adenosine triphosphate (ATP) production, which collectively mitigate tissue damage caused by hypoxia^1,2^. While the benefits of FDP in protecting hypoxic tissues have been well-documented in preclinical and clinical studies^3–5^, its interactions with the hemostatic system—particularly its effects on coagulation dynamics and platelet function—remain incompletely understood.

Consequently, elucidating the effects of FDP on hemostasis is of clinical significance to ensure its safe application in these vulnerable patients. FDP is frequently administered systemically to critically ill patients, a population that often presents with underlying coagulopathies and platelet dysfunction^6^. Thus, clarifying FDP’s effects on hemostasis is crucial for ensuring its safe use in such high-risk groups. Emerging evidence has suggested that FDP may modulate hemostatic processes^6,7^. For instance, our preliminary work demonstrated that FDP can alter results of conventional coagulation assays (prothrombin time [PT], activated partial thromboplastin time [APTT], thrombin time [TT]) and inhibit platelet aggregation^8^, indicating potential broad interference with components of the hemostatic system. However, prior studies have not systematically investigated FDP’s impact on the critical phase of clot initiation—a key determinant of bleeding or thrombotic risk—nor have they explored its combined effects on specific coagulation factors and platelet signaling pathways.

To address these limitations, the present study employed thromboelastography (TEG), a viscoelastic assay that comprehensively evaluates clot formation kinetics^9^, alongside quantitative analyses of coagulation factor activities and platelet aggregation. By using three distinct TEG platforms and assessing multiple coagulation factors and platelet agonists, this study aimed to characterize the in vitro effects of FDP on hemostasis, with a focus on its potential dual roles in coagulation and platelet function. Such insights are essential for refining clinical guidelines regarding FDP use and exploring its potential implications in anticoagulant therapy.

## Materials and methods

### Reagents and instruments

#### TEG systems

Maiketian Haema TX (Lot 20,231,101, Shenzhen Maiketian Biomedical Technology Co., Ltd., China); Lepu CFMS LEPU-8880 (with Thrombelastograph General Cup Test Kit, Lot 23SH0102, Lepu Medical Technology Co., Ltd., China); Dingrun DRNX-III (with Activated Coagulation Reagent, Lot 20,230,504, Chongqing Dingrun Medical Equipment Co., Ltd., China). TEG parameters measured: clot reaction time (R-time, min), clot formation time (K-time, min), α-angle (°), maximum amplitude (MA, mm). Reagent mechanism: Kaolin activator (negatively charged particles) initiated intrinsic coagulation via factor XII contact activation; calcium chloride (CaCl₂) promoted fibrin polymerization.

#### Coagulation factor assays

Sysmex CS5100 Coagulation Analyzer (Siemens, Germany) with screening reagents (APTT: Dade Actin Activated Cephaloplastin Reagent, Lot 562729 A; PT: Thromborel S, Lot 568,182), factor-deficient plasmas (factors II [Lot 503659], V [Lot 575712], VII [Lot 500776], VIII [Lot 560857 A], IX [Lot 504172B], X [Lot 504029], XI [Lot 503358B], XII [Lot 503427]), and controls (Standard Human Plasma, Lot 563,120; CONTROL N, Lot 507,936; CONTROL P, Lot 556,743).

#### Platelet aggregation agonists (Sysmex, Japan)

Adenosine diphosphate (ADP, 5 μmol/L), arachidonic acid (AA, 1 mmol/L), collagen (Col, 2.5 μg/mL), epinephrine (Epi, 10 μmol/L).

Test compound: Fructose-1,6-diphosphate (FDP, sodium salt; Anhui Weilman Pharmaceutical Co., Ltd., Lot 20,231,001, China).

### Study participants and laboratory procedures

#### Ethical compliance

 This study was approved by the Ethics Review Committee of Anhui No. 2 Provincial People’s Hospital (Approval [R] 2024–037). All participants provided written informed consent.

#### Quality control

 Before sample analysis, all assay systems were validated using manufacturer-provided controls (normal/abnormal ranges) to ensure reliability, alongside routine calibrations.

#### Participants

 Venous blood (14 mL) was collected from 11 healthy adult volunteers (6 males, 5 females; age 31–48 years) with no history of coagulopathies or anticoagulant use within 2 weeks. PRP was prepared from 5 additional healthy donors (3 males, 2 females; age 31–42 years; platelet counts of > 150 × 10⁹/L) using standardized protocols^10^ (centrifugation at 150 × *g* for 10 min at 22 °C).

The concentration range of FDP (0–6 mg/mL) used in this study was designed to encompass and exceed the estimated clinically relevant plasma concentration (1.81–3.71 mg/mL). This estimation was derived from typical clinical intravenous doses (5–10 g per 70 kg body weight; Anhui Weilman Pharmaceutical Co.), assuming complete and immediate distribution in the plasma volume. A preliminary experiment at 3.71 mg/mL confirmed significant biological effects, which justified the subsequent comprehensive concentration–response testing across the full 0–6 mg/mL range.

#### Experimental protocols: TEG

 Whole blood supplemented with FDP (0, 1, 2, 3, 4, 5, 6 mg/mL) was incubated at 37 °C for 1 h. R-time, K-time, α-angle, and MA were measured using Maiketian, Lepu, and Dingrun systems.

#### Coagulation factor axdxssays

 Plasma spiked with FDP (0–6 mg/mL) was incubated at 37 °C for 1 h. Activities of factors II, V, VII, VIII, IX, X, XI, and XII were quantified via Sysmex CS5100.

#### Platelet aggregation

 PRP supplemented with FDP (0–6 mg/mL) was stimulated with agonists (ADP, AA, collagen, epinephrine). Maximum aggregation rate (MA%) was recorded within 4 h of PRP preparation using Sysmex CS5100 software.

All samples were analyzed in triplicate within 2 h of preparation to minimize preanalytical variability.

### pH control experiments

To control for potential confounding effects arising from pH shifts due to the intrinsic acidity of FDP, additional experiments were performed. Venous blood was collected from 11 healthy volunteers using a 21-gauge needle and anticoagulated with 3.2% (0.109 M) trisodium citrate at a 1:9 (vol/vol) ratio. The first tube was discarded to avoid tissue thromboplastin contamination, and all samples were processed within one hour of collection. Platelet-rich plasma (PRP) and platelet-poor plasma (PPP) were prepared by centrifugation at 150 × *g* for 10 min and 2500 × *g* for 15 min, respectively, at room temperature (22 ± 2℃), in accordance with standard guidelines^11–13^. No exogenous calcium was added during light transmission aggregometry, consistent with CLSI, BSH, and ISTH recommendations^11–13^.

Each sample was divided into three experimental sets, each comprising seven aliquots (3 mL PET tubes) corresponding to FDP concentrations of 0–6 mg/mL.

Unbuffered FDP group: FDP stock solution (0.5 mg/μL) was added to achieve target concentrations without pH adjustment.

pH-matched acid control group: A minimal volume of 1 M HCl was added to match the pH of the corresponding unbuffered FDP aliquot, without FDP.

Buffered FDP group: FDP stock solution was pre-adjusted to pH 7.4 using 1 M NaOH before addition.

Key assays were repeated across these groups to differentiate pH-related artifacts from FDP-specific effects. Thromboelastography R-time was assessed using the Maiketian analyzer. Coagulation factor activities (Factors V, VII, IX, XI, XII) and platelet aggregation in response to ADP (5 μmol/L), AA (1 mmol/L), collagen (2.5 μg/mL), and epinephrine (10 μmol/L) were measured using a Sysmex CS5100 system. All procedures followed manufacturer protocols, and the stability of reagents was verified before use.

### Statistical analysis

Samples with 0 mg/mL FDP served as baseline controls. Percentage changes relative to controls were computed to quantify FDP-induced alterations. Statistical analyses were performed using SPSS 20.0 (IBM, Armonk, NY, USA) and Microsoft Excel for data organization.

Pearson's correlation analysis evaluated associations between FDP concentrations (0–6 mg/mL) and TEG parameters (R-time, K-time, α-angle, MA) or coagulation factor activities.

For the analysis of platelet aggregation data across multiple FDP concentrations, one-way repeated measures ANOVA was employed, followed by Dunnett’s post-hoc test for comparisons between each FDP concentration (1–6 mg/mL) and the baseline control (0 mg/mL). This approach controls for the increased risk of Type I error associated with multiple comparisons.

Statistical significance was defined as *P* < 0.05 (two-tailed). A supplementary non-parametric analysis (Spearman's correlation and Friedman test with Dunn’s post-hoc test) was also performed, which confirmed all conclusions derived from the parametric tests.

## Results

### FDP concentration-dependently prolongs TEG clot reaction time

TEG analysis across three platforms (Maiketian, Lepu, Dingrun) showed that FDP prolonged R-time in a concentration-dependent manner (0–6 mg/mL). Strong positive correlations were observed between FDP concentrations and R-time (Table 1):

**Table 1:**
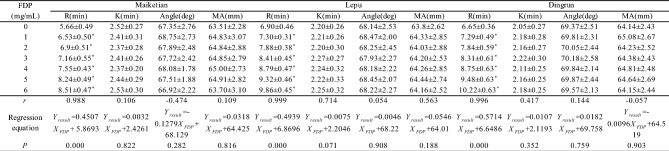
In vitro thromboelastography parameters of pooled blood at different FDP concentrations: The coagulation reaction time (R, min), coagulation time (K, min), maximum amplitude (MA, mm), and α-angle(data from three TEG systems; *x̄* ± *s*, *n* = 11). TEG system Maikertian (China), Lepu (china), Dingrun (China). *p < 0.001 vs. 0 mg/mL FDP for all parameters (paired t-test).

Maiketian:* r* = 0.988 (*P* < 0.001), *Y* = 0.4507*X* + 5.8693.

Lepu:* r* = 0.999 (*P* < 0.001), *Y* = 0.4939*X* + 6.8696.

Dingrun: *r* = 0.996 (*P* < 0.001), *Y* = 0.5714*X* + 6.6486.

In contrast, no significant correlations were observed between FDP concentrations and other TEG parameters (K-time, α-angle, MA; all *P* > 0.05). These findings indicate FDP selectively impairs the coagulation initiation phase (reflected by R-time) but not clot propagation, kinetics, or strength.

### FDP dose-dependently inhibits intrinsic and extrinsic pathway coagulation factors

FDP selectively inhibited coagulation initiation factors in a concentration-dependent manner (0–6 mg/mL). Significant negative correlations were observed between FDP concentrations and activities of intrinsic/extrinsic pathway factors (Table 2):

**Table 2:** Coagulation factors (II, V, VII, VIII, IX, X, XI, XII) activities in pooled plasma with different FDP concentrations in vitro (x̄ ± s, n = 11).

FDP (mg/mL)	Coagulation factor activities(%)							
	V	VII	IX	XI	XII	II	VIII	X
0	125.25 ± 18.96	146.85 ± 26.42	109.35 ± 9.07	75.88 ± 10.15	68.24 ± 7.40	130.05 ± 24.54	112.26 ± 17.50	121.57 ± 17.69
1	123.14 ± 19.33*	144.94 ± 25.13*	107.25 ± 8.58*	74.44 ± 10.08*	66.85 ± 7.48*	128.16 ± 22.13	111.47 ± 17.27	120.27 ± 20.73
2	121.99 ± 18.83*	143.22 ± 24.67*	104.18 ± 8.31*	72.42 ± 10.09*	65.93 ± 7.52*	126.95 ± 22.12	110.88 ± 17.87	123.57 ± 19.10
3	119.66 ± 17.16*	141.89 ± 24.15*	101.83 ± 7.80*	71.30 ± 10.30*	64.95 ± 7.25*	127.66 ± 22.27	112.05 ± 17.60	121.68 ± 20.00
4	118.97 ± 17.29*	139.80 ± 24.47*	99.85 ± 7.04*	70.41 ± 10.05*	64.25 ± 7.51*	126.44 ± 23.66	111.01 ± 16.85	123.98 ± 17.98
5	116.26 ± 16.46*	138.05 ± 23.52*	98.70 ± 7.00*	69.07 ± 9.87*	62.91 ± 7.68*	127.23 ± 23.57	111.76 ± 18.21	123.12 ± 17.22
6	114.65 ± 16.38*	136.47 ± 22.38*	97.29 ± 7.10*	67.49 ± 9.06*	61.47 ± 7.35*	127.05 ± 22.51	109.38 ± 17.13	122.25 ± 17.13
*r*	−0.995	−0.99	−0.989	−0.997	−0.995	−0.737	−0.630	0.484
Regression equation	*Y*_*result*_ = −1.735*X*_*FDP*_ + 125.19	*Y*_*result*_ = −1.7264*X*_*FDP*_ + 146.78	*Y*_*result*_ = −2.0575*X*_*FD*P_ + 108.81	*Y*_*result*_ = −1.3543*X*_*FDP*_ + 75.636	*Y*_*result*_ = −1.0668*X*_*FDP*_ + 68.143	*Y*_*result*_ = −0.4061*X*_*FDP*_ + 128.87	*Y*_*result*_ = −0.2832*X*_*FDP*_ + 112.11	*Y*_*result*_ = 0.2911*X*_*FDP*_ + 121.48
*P*	< 0.001	< 0.001	< 0.001	< 0.001	< 0.001	0.059	0.129	0.27

Significant differences versus the control group (0 mg/mL FDP) were observed (*P < 0.05).

Factor V:* r* = − 0.995 (*P* < 0.001).

Factor VII: *r* = − 0.990 (*P* < 0.001).

Factor IX:* r* = − 0.989 (*P* < 0.001).

Factor XI: *r* = − 0.997 (*P* < 0.001).

Factor XII: *r* = − 0.995 (*P* < 0.001).

In contrast, the activities of common pathway factors (II, VIII, and X) showed no statistically significant alterations after FDP treatment (*P* > 0.05; Table 3). Conversely, the activities of factors V, VII, IX, XI, and XII were significantly inhibited (*P* < 0.001). At the clinically relevant concentration of 3.71 mg/mL FDP, the clot reaction time (R-time) measured by the Maiketian thromboelastography system was significantly prolonged from 5.36 ± 0.38 min to 7.28 ± 0.31 min (mean ± SD, *P* < 0.001), representing an increase of approximately 35.8% compared to the baseline control (Table 3). Notably, despite the variable extent of inhibition in the activities of the initiation factors (ranging from approximately 3% to 15% reduction at 3.71 mg/mL), the observed pronounced prolongation in R-time underscores a functionally significant anticoagulant effect at this clinically relevant dose.

**Table 3:** Effects of sodium FDP (3.71 mg/mL FDP) on coagulation factor activity and TEG parameters(*n *= 11).

Sample	R(min)		V(%)		VII(%)		IX(%)		XI(%)		XII(%)		II(%)		VIII(%)		X(%)	
	Before	After	Before	After	Before	After	Before	After	Before	After	Before	After	Before	After	Before	After	Before	After
	Treatment	Treatment	Treatment	Treatment	Treatment	Treatment	Treatment	Treatment	Treatment	Treatment	Treatment	Treatment	Treatment	Treatment	Treatment	Treatment	Treatment	Treatment
1	5.53	7.30	118.5	117.8	140.1	137.4	99.5	96.5	77.8	73.5	65.5	61.3	124.5	123.4	109.8	111.2	119.7	123.7
2	5.13	7.12	125.7	115.7	146.6	144.3	87.4	85.4	76.5	71.4	64.4	65.4	121.5	125.5	112.3	109.9	118.9	127.4
3	5.65	6.88	134.4	123.5	152.1	144.5	87.8	81.4	67.6	68.9	58.0	56.5	132.5	124.5	102.7	109.7	129.7	125.5
4	5.43	7.23	113.5	109.5	134.5	125.5	99.4	89.9	79.5	73.5	68.5	56.7	123.4	111.5	113.4	116.7	125.5	129.6
5	4.95	7.34	112.6	108.6	138.6	135.6	104.4	96.4	81.4	69.5	56.7	54.3	120.4	119.5	112.4	109.7	123.5	116.7
6	5.99	7.88	117.9	112.5	145.6	144.4	87.9	88.9	67.4	65.3	71.4	64.6	119.5	121.4	107.6	102.4	129.1	123.5
7	4.89	6.56	114.2	105.5	151.4	145.3	94.1	81.4	64.6	57.4	71.4	67.5	118.5	117.8	109.8	109.8	127.5	131.4
8	5.67	7.23	132.5	124.6	155.3	150.4	99.4	90.5	69.7	60.6	61.8	55.8	132.7	134.5	113.3	112.5	119.6	127.6
9	5.61	7.77	132.4	126.6	145.5	140.3	96.3	92.7	72.4	67.5	63.5	58.8	128.4	125.6	106.4	103.3	113.5	115.5
10	4.88	7.13	122.3	119.4	146.3	141.3	105.4	104.5	74.5	72.5	65.7	56.7	117.7	118.5	104.9	105.9	115.7	116.8
11	5.21	7.67	133.4	132.4	139.3	136.6	112.1	104.5	71.8	67.4	63.8	61.5	114.6	113.4	113.7	109.5	116.6	115.0
*t*	17.15		5.34		6.22		4.42		4.69		4.48		1.19		0.48		0.46	
*P*	< 0.001		< 0.001		< 0.001		< 0.001		< 0.001		< 0.001		0.26		0.64		0.77	

Thromboelastography reaction time (R), reflecting coagulation initiation, was measured using a TEG system (Maiketian, China). Coagulation factor activity (%) was determined with a CS-5100 automated coagulation analyzer (Sysmex, Japan). Statistical analysis revealed significant differences between after-treatment (3.71 mg/mL FDP) and before-treatment (0 mg/mL FDP) measurements (*P* < 0.01). Given the consistent prolongation of R-time observed across all three TEG platforms (Table 1), the Maiketian system was selected for further detailed analysis of FDP effects at the clinically relevant concentration of 3.71 mg/mL.

Despite these modest reductions in factor activities (< 10% at the therapeutic concentration of 3.71 mg/mL), the concomitant prolongation of R-time exceeded 20% across all TEG platforms. This discrepancy suggests that even subtle inhibition of multiple initiation factors can synergistically lead to a functionally significant anticoagulant effect at clinically relevant doses.

### FDP dose-dependently inhibits platelet aggregation

**Fig. 1:**
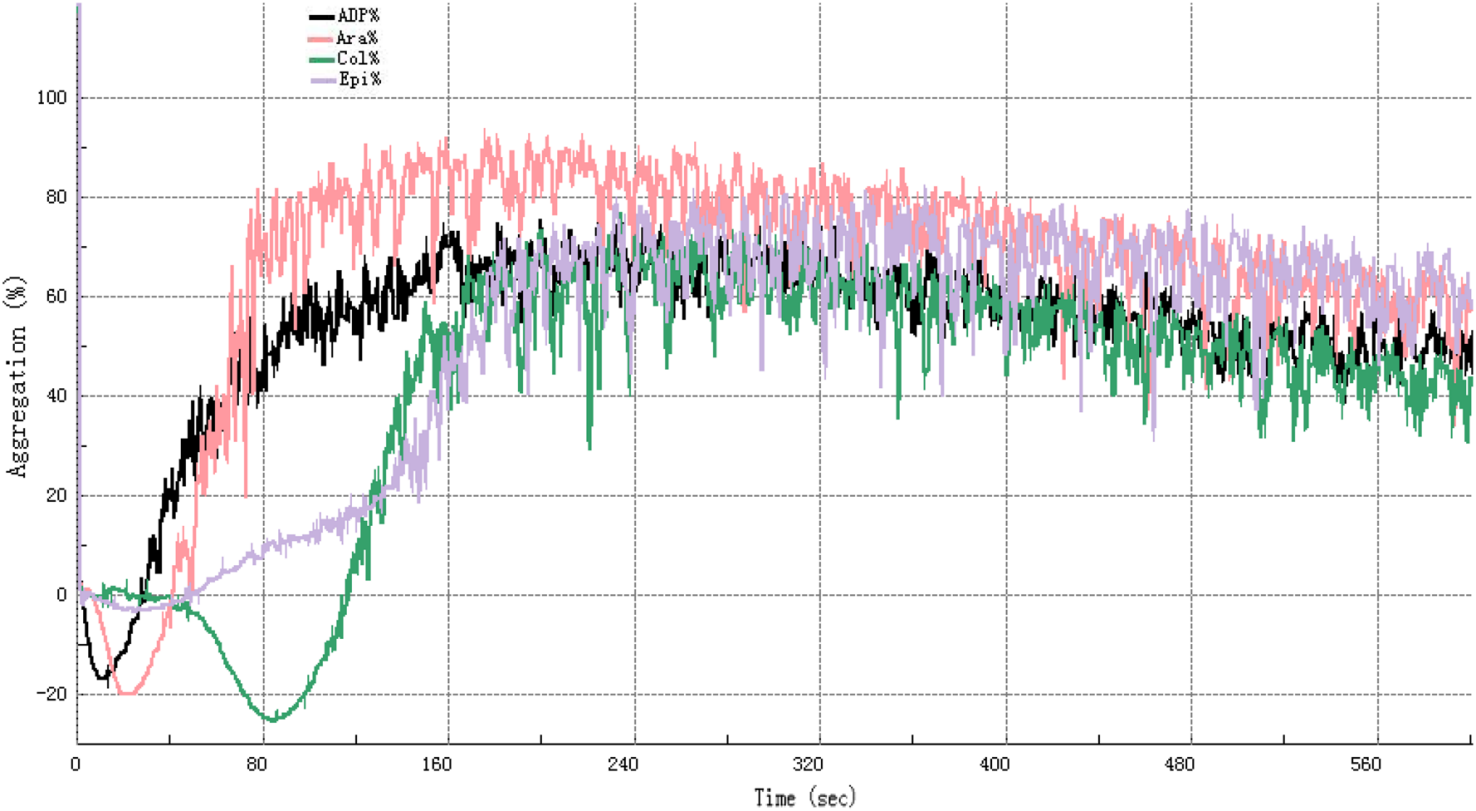
Representative curves depicting the temporal changes in platelet aggregation rate (%) in platelet-rich plasma following stimulation with various agonists (5 μmol/L ADP, 1 mmol/L AA, 2.5 μg/mL collagen, and 10 μmol/L epinephrine), as monitored by the Sysmex CS-5100 automated coagulation analysis system.

The time and aggregation rate changes of the sample platelets in response to different agonists are shown in Fig. 1. FDP dose-dependently inhibited platelet aggregation induced by all agonists tested (Table 4). The maximum aggregation rates (MA%) decreased progressively with increasing FDP concentrations (0–6 mg/mL). Specifically, at the highest concentration of 6 mg/mL FDP, MA% was markedly reduced to 6.02 ± 2.67% for ADP (*P* < 0.001), 5.86 ± 2.73% for epinephrine (*P* < 0.001), 59.08 ± 4.86% for arachidonic acid (AA,* P* < 0.01), and 62.6 ± 7.25% for collagen (P < 0.001), compared to their respective baseline controls (0 mg/mL FDP).

**Table 4:**
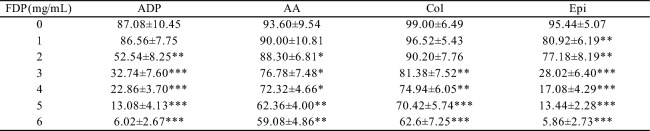
Maximum aggregation rate (MA%) of platelets in platelet-rich plasma from healthy adults treated with different FDP concentrations in vitro (determined using Sysmex CS5100 coagulation analyzer; *x̄* ± s,* n *= 5). *Significant differences versus baseline (0 mg/mL FDP) were observed (paired student’s t-test): *p < 0.005, **p < 0.01, ***p < 0.001.

The sensitivity to FDP inhibition and the onset of significant suppression varied among the agonists. Epinephrine-induced aggregation was the most sensitive, with significant inhibition (*P* < 0.01) first observed at 1 mg/mL FDP. In contrast, significant suppression of ADP-induced aggregation commenced at 2 mg/mL (*P* < 0.01), while AA and collagen-induced aggregation required higher concentrations, with significant effects appearing at 3 mg/mL (*P* < 0.05) and 5 mg/mL (*P* < 0.001), respectively.

Notably, near-complete inhibition of aggregation (MA% < 10%) was achieved for the ADP and epinephrine pathways at FDP concentrations ≥ 5 mg/mL. While arachidonic acid and collagen pathways also exhibited significant, dose-dependent suppression, the extent of inhibition was less profound compared to ADP and epinephrine. These results demonstrate the potent and broad-spectrum, yet agonist-dependent, antiplatelet properties of FDP.

### pH control experiments confirm FDP-specific effects on hemostasis

To definitively determine whether the observed anticoagulant and antiplatelet effects of FDP were attributable to pH changes resulting from its intrinsic acidity, we performed a series of well-controlled experiments under three distinct conditions: unbuffered FDP, FDP buffered to physiological pH (7.4), and an acid control (pH-matched using HCl, without FDP).

**Fig. 2:**
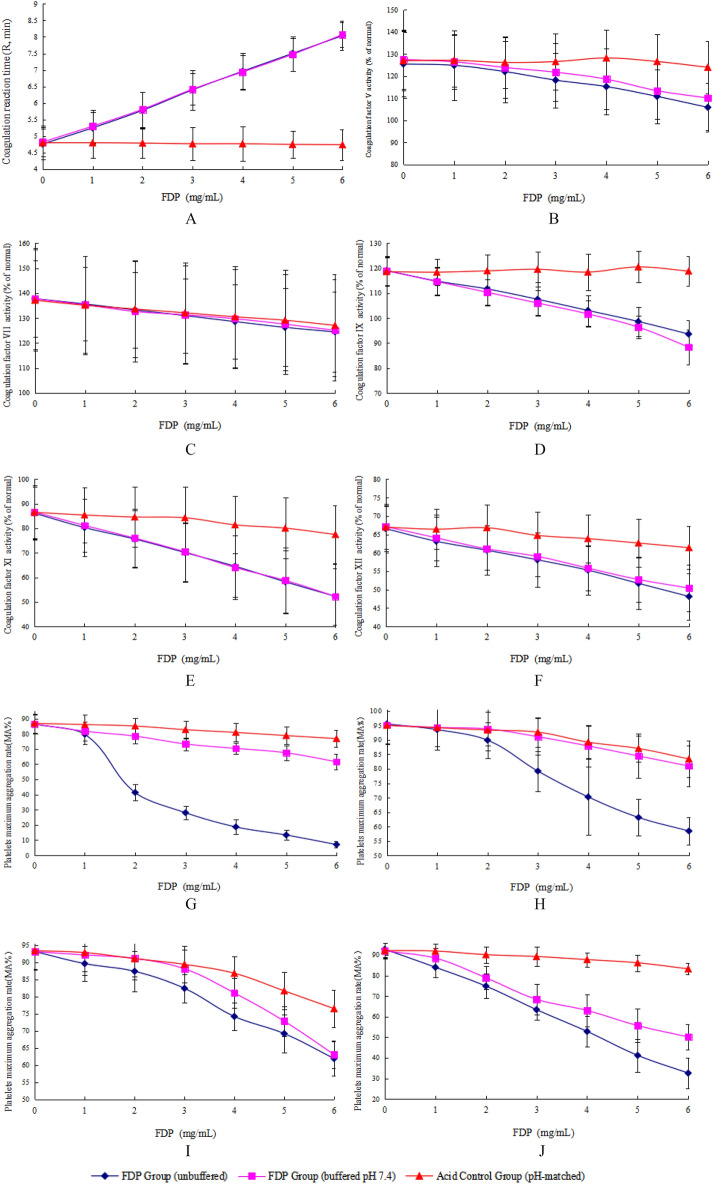
(**A**) Coagulation reaction time (R, min) under different FDP concentrations and conditions (*x̄* ± *s*,* n *= 11). (**B**) Coagulation factor V activity (% of normal) under different FDP concentrations and conditions ($$\overset{\lower0.5em\hbox{$\smash{\scriptscriptstyle\frown}$}}{x}$$±* s*, *n *= 11). (**C**) Coagulation factor VII activity (% of normal) under different FDP concentrations and conditions ($$\overset{\lower0.5em\hbox{$\smash{\scriptscriptstyle\frown}$}}{x}$$ ± *s,* *n* = 11). (**D**) Coagulation factor IX activity (% of normal) under different FDP concentrations and conditions ($$\overset{\lower0.5em\hbox{$\smash{\scriptscriptstyle\frown}$}}{x}$$ ± *s*, *n* = 11). (**E**) Coagulation factor XI activity (% of normal) under different FDP concentrations and conditions ($$\overset{\lower0.5em\hbox{$\smash{\scriptscriptstyle\frown}$}}{x}$$ ±* s, n *= 11). (**F**) Coagulation factor XII activity (% of normal) under different FDP concentrations and conditions ($$\overset{\lower0.5em\hbox{$\smash{\scriptscriptstyle\frown}$}}{x}$$ ±* s*, *n *= 11). (**G**) Maximum aggregation rate (MA, %) of platelets induced by ADP under different FDP concentrations and conditions ($$\overset{\lower0.5em\hbox{$\smash{\scriptscriptstyle\frown}$}}{x}$$ ± *s*, *n *= 11). (**H**) Maximum aggregation rate (MA, %) of platelets induced by AA under different FDP concentrations and conditions ($$\overset{\lower0.5em\hbox{$\smash{\scriptscriptstyle\frown}$}}{x}$$ ± *s*, *n* = 11). (**I**) Maximum aggregation rate (MA, %) of platelets induced by Col under different FDP concentrations and conditions ($$\overset{\lower0.5em\hbox{$\smash{\scriptscriptstyle\frown}$}}{x}$$±* s*,* n* = 11). (**J**) Maximum aggregation rate (MA, %) of platelets induced by Epi under different FDP concentrations and conditions($$\overset{\lower0.5em\hbox{$\smash{\scriptscriptstyle\frown}$}}{x}$$ ±* s, n *= 11).

Thromboelastography R-Time (Fig. 2A): FDP concentration-dependently prolonged the R-time in both unbuffered and pH-buffered groups. For example, at 6 mg/mL FDP, R-time increased to 8.04 ± 0.44 min (unbuffered) and 8.07 ± 0.38 min (buffered). In contrast, the acid control group showed no significant change across the same concentration range (4.74–4.80 min), indicating that the prolongation of clot initiation is a specific effect of FDP, independent of pH.

Coagulation Factor Activities (Fig. 2B–F): A dose-dependent inhibition of coagulation factors V, VII, IX, XI, and XII was observed in both unbuffered and pH-buffered FDP groups, with strong negative correlations (all P < 0.001) between FDP concentration and factor activity. For instance, Factor XI activity decreased from 86.13% to 52.24% (unbuffered) and from 86.62% to 52.23% (buffered) over 0–6 mg/mL FDP. The acid control group, however, exhibited only minor and non-systematic variations (e.g., Factor XI: 86.49% to 77.45%), confirming that the inhibition is specifically mediated by FDP and not by low pH.

Platelet Aggregation (Fig. 2G–J): FDP significantly suppressed platelet aggregation in response to all agonists (ADP, AA, collagen, and epinephrine) in a dose-dependent manner. While the unbuffered FDP group showed pronounced inhibition, the pH-buffered group also exhibited significant suppression, though to a lesser extent for certain agonists. For example, ADP-induced aggregation at 6 mg/mL FDP was reduced to 7.11% (unbuffered) versus 61.56% (buffered), indicating that the strong inhibition in the unbuffered setting is partly pH-dependent. Nevertheless, the residual suppression in the buffered group (61.56% vs. 86.52% baseline) confirms an additional FDP-specific antiplatelet effect. Similar trends were observed for other agonists, though the magnitude of pH buffering varied.

In summary, these controlled experiments demonstrate that the core anticoagulant effects of FDP—specifically the prolongation of clot initiation and inhibition of key coagulation factors—are intrinsic to FDP and not secondary to pH changes. The antiplatelet effects, while modulated by pH under certain agonist conditions, also include a significant FDP-specific component.

## Discussion

This study systematically demonstrates that FDP exerts dual anticoagulant and antiplatelet effects in vitro, characterized by selective inhibition of coagulation initiation factors, prolonged clot initiation, and broad suppression of platelet aggregation. These findings provide novel insights into FDP’s uncharacterized hemostatic properties, with implications for clinical safety and potential therapeutic expansion.

### FDP selectively impairs coagulation initiation

TEG revealed FDP concentration-dependently prolonged R-time across three platforms, while sparing other parameters. R-time, a critical marker of the coagulation initiation phase, reflects the time required for initial fibrin clot formation via intrinsic and extrinsic pathway activation^9,14^. Our observation of R-time prolongation (21.8–48.3% at therapeutic concentrations) aligns with selective reductions in activities of factors V, VII, IX, XI, and XII, highlighting FDP’s targeted interference with coagulation initiation.

Factor VII, a key trigger of the extrinsic pathway (activated by tissue factor), and factors IX, XI, XII (intrinsic pathway components) are pivotal for amplifying coagulation cascades^15,16^. The robust negative correlations between FDP levels and these factors (*r* = − 0.989 to − 0.997) suggest a direct or indirect inhibitory interaction. Notably, factors II, VIII, and X—central to the common pathway—remained unaffected, indicating FDP does not disrupt late-stage fibrin formation or thrombin generation.

This selectivity distinguishes FDP from conventional anticoagulants: heparin broadly inhibits thrombin and factor Xa via antithrombin III^17–19^, while warfarin non-selectively reduces vitamin K-dependent factors (II, VII, IX, X)^20^. FDP’s mechanism may involve competitive binding to coagulation factors or modulation of their conformational activation, though further structural biology studies are needed to clarify this.

The discrepancy in R-time prolongation across TEG platforms (Lepu > Dingrun > Maiketian) likely stems from differences in reagent compositions (e.g., kaolin activation efficiency)^9^, emphasizing the need for platform-specific reference ranges when interpreting FDP’s effects.

### Potent antiplatelet effects: broad agonist inhibition and the role of pH

Our data confirm that FDP dose-dependently suppresses platelet aggregation induced by ADP, AA, collagen, and epinephrine. The critical insights from our pH-controlled experiments allow us to refine the interpretation of this effect. pH-control experiments allowed us to delineate the contributions of FDP itself versus acidity. A substantial FDP-specific antiplatelet component was confirmed, as significant suppression—though variably attenuated—persisted in the pH-buffered group for all agonists compared to their pH-matched controls. However, the results also reveal a more nuanced picture: the dramatic attenuation of inhibition in the buffered group, particularly for ADP-induced aggregation (e.g., from ~ 93% inhibition in unbuffered to ~ 29% inhibition at pH 7.4 at 6 mg/mL), indicates that the acidic milieu is the primary driver of the full antiplatelet potency for this particular pathway, likely by profoundly disrupting intracellular signaling.

These findings extend prior reports: Cavallini et al. first observed FDP-mediated inhibition of platelet activation in 1992^6^, de Oliveira et al. demonstrated reduced ADP-induced aggregation in septic rats treated with FDP^7^, and epinephrine induces aggregation by amplifying agonist signals and potentiating the GPVI pathway via α2A-adrenergic receptors^21^. Our study expands this by showing broad efficacy across agonists, indicating FDP may target shared downstream pathways (e.g., glycoprotein IIb/IIIa activation or intracellular Ca^2^⁺mobilization) rather than agonist-specific receptors. For instance, AA-induced aggregation relies on cyclooxygenase-1 (COX-1) and thromboxane A₂ synthesis^22^, while collagen activates glycoprotein VI^23^; FDP’s inhibition of both suggests a converging regulatory point, possibly related to energy metabolism. As a key glycolytic intermediate, FDP is known to enhance cellular ATP production^1,5^. Since platelet function is critically dependent on ATP levels^24,25^, it would be valuable to investigate whether FDP influences platelet activity by modulating ATP balance or related signaling pathways.

### Clinical context: balancing cytoprotection and hemostatic risk

FDP is clinically used for ischemic conditions due to its cytoprotective roles in ATP preservation and anti-inflammatory effects^2–4^. Our data raise concerns about its hemostatic safety in high-risk populations: patients with coagulation factor deficiencies (e.g., factor VII or IX deficiency) or concurrent antithrombotic therapy (e.g., aspirin, which inhibits AA-mediated aggregation) may experience exacerbated bleeding^26^. For example, in perioperative patients with acquired coagulopathy, FDP-induced R-time prolongation at therapeutic doses may amplify hemostatic dysfunction.

Conversely, the dual anticoagulant and antiplatelet profile of FDP suggests intriguing therapeutic potential. Unlike single-target agents (e.g., clopidogrel^26^, rivaroxaban^27^), FDP simultaneously modulates coagulation initiation and platelet aggregation, which could be advantageous in thrombotic disorders characterized by multi-pathway activation. However, the substantial limitations of our in vitro study must be considered, as the in vivo applicability, safety profile, and net hemostatic impact of FDP require rigorous evaluation in physiological contexts. Factors such as plasma protein binding, metabolic conversion, and endothelial interactions may significantly alter FDP’s activity. Therefore, any consideration of FDP for antithrombotic therapy is highly speculative and must be preceded by extensive in vivo studies to delineate its therapeutic window and rule out unacceptable bleeding risks.

### Limitations and future directions

This study has limitations: (1) The in vitro design cannot replicate in vivo complexities (e.g., endothelial interactions, blood flow dynamics). (2)The initial sample size for platelet aggregation assays (n = 5) was relatively small, which, while common in preliminary in vitro studies, limits the generalizability of the initial findings. To address this and specifically control for pH confounding effects, we repeated key platelet aggregation measurements, along with TEG R-time and coagulation factor activities, using a larger cohort (n = 11) under pH-controlled conditions (Fig. 2A–J). The consistency of the results across the expanded sample reinforces the dose-dependent inhibitory effect of FDP. (3) The mechanism underlying factor selectivity and platelet inhibition remains speculative; structural studies (e.g., FDP-coagulation factor binding assays) and proteomic analyses of FDP-treated platelets could provide clarity.(4) Furthermore, employing advanced platforms that simulate physiological flow and shear stress, such as the Total Thrombus-formation Analysis System (T-TAS), would be invaluable. This approach could provide a more comprehensive and physiologically relevant evaluation of FDP’s impact on the dynamic interplay between platelets, coagulation factors, and thrombus formation under flow conditions.

Future research should focus on: (1) In vivo models (e.g., murine thrombosis/bleeding models) to confirm FDP’s hemostatic effects. (2) Clinical studies evaluating bleeding risk in FDP-treated patients with coagulopathies. (3) Exploration of FDP’s therapeutic window in thrombotic conditions, potentially in combination with existing antithrombotics.

## Conclusions

In summary, this in vitro study demonstrates that FDP exerts dual anticoagulant and antiplatelet effects. These are characterized by a concentration-dependent prolongation of clot initiation (reflected by TEG R-time) through selective inhibition of coagulation initiation factors (V, VII, IX, XI, and XII), alongside a broad suppression of platelet aggregation induced by multiple agonists. The selective targeting of coagulation initiation phases and platelet aggregation distinguishes FDP from conventional antithrombotic agents, highlighting its unique pharmacological profile. However, caution is warranted when extrapolating these in vitro findings to clinical scenarios, particularly in high-risk populations where FDP may exacerbate bleeding risks. Further studies are required to validate these findings in vivo, clarify the underlying molecular mechanisms, and evaluate the clinical implications of FDP’s dual hemostatic effects, which will be critical for balancing its cytoprotective benefits against its newly identified impacts on hemostasis.

## Data Availability

The original contributions presented in this study are included in the article. The raw data supporting the conclusions will be made available by the corresponding author, Tongqing Chen, without restriction. Requests for raw data can be directed to Tongqing Chen via email: Tongqingchen@126.com.
